# Persistent Environmental Pollutants and Couple Fecundity: The LIFE Study

**DOI:** 10.1289/ehp.1205301

**Published:** 2012-11-14

**Authors:** Germaine M. Buck Louis, Rajeshwari Sundaram, Enrique F. Schisterman, Anne M. Sweeney, Courtney D. Lynch, Robert E. Gore-Langton, José Maisog, Sungduk Kim, Zhen Chen, Dana B. Barr

**Affiliations:** 1 Division of Epidemiology, Statistics and Prevention Research, *Eunice Kennedy Shriver* National Institute of Child Health and Human Development, National Institutes of Health, Department of Health and Human Services, Rockville, Maryland, USA; 2 Department of Epidemiology and Biostatistics, School of Rural Public Health, Texas A & M Health Science Center, College Station, Texas, USA; 3 Department of Obstetrics and Gynecology, College of Medicine, The Ohio State University, Columbus, Ohio, USA; 4 The EMMES Corporation, Rockville, Maryland, USA; 5 Department of Occupational and Environmental Health, Rollins School of Public Health, Emory University, Atlanta, Georgia, USA

**Keywords:** conception, cotinine, fecundity, organochlorine pesticides, polybrominated diphenyl ethers, polychlorinated biphenyls, perfluorochemicals, time to pregnancy

## Abstract

Background: Evidence suggesting that persistent environmental pollutants may be reproductive toxicants underscores the need for prospective studies of couples for whom exposures are measured.

Objectives: We examined the relationship between selected persistent pollutants and couple fecundity as measured by time to pregnancy.

Methods: A cohort of 501 couples who discontinued contraception to become pregnant was prospectively followed for 12 months of trying to conceive or until a human chorionic gonadotrophin (hCG) test confirmed pregnancy. Couples completed daily journals on lifestyle and provided biospecimens for the quantification of 9 organochlorine pesticides, 1 polybrominated biphenyl, 10 polybrominated diphenyl ethers, 36 polychlorinated biphenyls (PCBs), and 7 perfluorochemicals (PFCs) in serum. Using Cox models for discrete time, we estimated fecundability odds ratios (FORs) and 95% CIs separately for each partner’s concentrations adjusting for age, body mass index, serum cotinine, serum lipids (except for PFCs), and study site (Michigan or Texas); sensitivity models were further adjusted for left truncation or time off of contraception (≤ 2 months) before enrollment.

Results: The adjusted reduction in fecundability associated with standard deviation increases in log-transformed serum concentrations ranged between 18% and 21% for PCB congeners 118, 167, 209, and perfluorooctane sulfonamide in females; and between 17% and 29% for *p,p´-*DDE and PCB congeners 138, 156, 157, 167, 170, 172, and 209 in males. The strongest associations were observed for PCB 167 (FOR 0.79; 95% CI: 0.64, 0.97) in females and PCB 138 (FOR = 0.71; 95% CI: 0.52, 0.98) in males.

Conclusions: In this couple-based prospective cohort study with preconception enrollment and quantification of exposures in both female and male partners, we observed that a subset of persistent environmental chemicals were associated with reduced fecundity.

The impact of persistent environmental chemicals on human reproduction is a topic of considerable interest. Although several persistent environmental chemicals or their metabolites have been detected in semen, follicular fluid, and genital tract fluid (De Felip et al. 2004; Jirsová et al. 2010; Wagner et al. 1990), questions remain about their bioavailability and ability to affect the series of highly interrelated and timed processes underlying successful human reproduction. Experimental and human evidence suggests that persistent environmental contaminants may be associated with reduced follicle count, altered estrous or menstrual cycles, ovulation inhibition, and increased pregnancy loss and resorption in humans and animals, respectively (Buck Louis et al. 2011b; Lione 1988; Nicolopoulou-Stamati and Pitsos 2001; Pocar et al. 2003; Torf et al. 2004).

To our knowledge, only one previous cohort study measured serum persistent organochlorine pollutants (POPs) in women who were recruited prior to conception and followed through 12 observed menstrual cycles (Buck Louis et al. 2009). However, there is evidence suggesting a reduction in female fecundity, as measured by a longer time to pregnancy (TTP), associated with persistent environmental chemicals such as 1,1-dichloro-2,2-bis(*p*-chlorophenyl)ethylene (DDE), dioxin, perfluorochemicals (PFCs), polybrominated diphenyl ethers (PBDEs), and polychlorinated biphenyls (PCBs) (Axmon et al. 2005; Eskenazi et al. 2010; Fei et al. 2009; Gesink Law et al. 2005; Harley et al. 2010) when measured in female partners. In general, previous studies have quantified exposures in women at varying times during pregnancy instead of measuring exposures during the critical preconception window (Bloom et al. 2009). Studies of pregnant women systematically exclude women who are unable to become pregnant, who may be the most heavily exposed. In addition, studies of pregnant women rely on retrospectively reported TTP, which has been shown to result in both under- and overreporting of TTP (Cooney et al. 2009).

Against a background of speculation that human fecundity may be declining (Lutz et al. 2003; Skakkebaek et al. 2001), possibly as a result of effects of environmental factors on both partners of the couple as well as lifestyle changes, we designed the Longitudinal Investigation of Fertility and the Environment (LIFE) Study of persistent environmental chemicals and couple fecundity. By design, we sought to explore a spectrum of persistent environmental chemicals measured in both partners in relation to couple fecundability, consistent with the couple-dependent nature of human reproduction (Buck Louis 2011).

## Methods

*Study design and cohort*. In the LIFE Study we used a prospective cohort design with preconception recruitment of couples who were discontinuing contraception for the purpose of becoming pregnant. The cohort, sampled from an enumerated target population, comprised couples of reproductive age who resided in specific geographic counties in Michigan or Texas with reported environmental exposure to persistent environmental chemicals, and who were planning pregnancy in the next 6 months. Given the absence of established sampling frameworks for recruiting couples who were planning pregnancy, we used a commercially available marketing database and a fishing/hunting license registry to recruit 501 couples during 2005–2007 from the counties in the two states. Inclusion criteria were as follows: *a*) females were 18–40 years of age and males were ≥ 18 years of age; partners were in a committed relationship; neither partner was medically/surgically sterile; the female’s menstrual cycle was between 21 and 42 days; the female received no injectable contraceptives within 12 months; the couple had not used contraception for < 2 months; and both partners were able to communicate in English or Spanish. Introductory letters were mailed to the targeted cohort (*n* = 424,423); after telephone screening to identify eligible couples (*n* = 1,184), 501 couples (42%) were enrolled (Buck Louis et al. 2011c).

*Data and biospecimen collection*. All data collection occurred in the couples’ home. At the start of the study visit, the female partner provided a urine sample that was tested with a home pregnancy test capable of detecting 25 mIU/mL human chorionic gonadotropin (hCG) to ensure she was not pregnant. This important step permitted us to differentiate between couples achieving pregnancy in the first few weeks after enrollment (i.e., with no menstrual cycle occurring between enrollment and pregnancy) versus during the first fully observed cycle, which we denote in the analysis as cycles 0 and 1, respectively. Each partner of the couple was interviewed separately by one of two research assistants. Interviews were followed by a standardized physical anthropometric assessment (Lohman et al. 1988) to determine body mass index (BMI), and blood and urine samples were collected. Specifically, ≈ 20 cc of nonfasting blood and ≈ 120 cc of urine were collected from each partner of the couple. Blood collection equipment was free of the contaminants under study. Couples were instructed how to complete daily journals regarding sexual intercourse and lifestyle factors (e.g., cigarette smoking); journals of females also recorded menstruation and results of home pregnancy tests. Couples had the option of completing journals either in hardcopy or online.

Female partners were instructed in the use of the Clearblue® Easy home fertility monitor (Swiss Precision Diagnostics formerly Unipath). This urine-based monitor tracks the rise in estrone-3-glucuronide (E_3_G) and luteinizing hormone (LH) across the follicular phase of the ovarian cycle, and displays a prompt that ranges from low to peak fertility to help the couple time intercourse relative to ovulation. The monitor was used to enhance couples’ ability to conceive, given that it is 99% accurate in detecting the LH surge, compared with the gold standard of vaginal ultrasonology (Behre et al. 2000). The monitor was intended to minimize each couple’s chances of missing ovulation, which might erroneously lengthen TTP. Women were also trained in the use and interpretation of the Clearblue® Easy home pregnancy test, a digital device that indicates the test result as either pregnant or not pregnant. All participants were remunerated $75 for full participation in the study. Approval for use of human subjects was obtained from all collaborating institutions, and all participants gave informed consent before participation.

*Toxicologic analysis*. All analyses were conducted by the Division of Laboratory Sciences, National Center for Environmental Health, Centers for Disease Control and Prevention, using established protocols for the quantification of persistent environmental chemicals in serum. Chemicals included *a*) 1 polybrominated biphenyl (PBB 153); *b*) 9 organochlorine pesticides [OCPs; hexachlorobenzene (HCB), β-hexachlorocyclohexane (β-HCH), γ-hexachlorocyclohexane (γ-HCH), oxychlordane, *trans*-nonachlor, *p,p´-*DDT, *o,p´*-DDT, *p,p´-*DDE, and mirex]; *c*) 10 PBDEs (congeners 17, 28, 47, 66, 85, 99, 100, 153, 154, and 183); *d*) 36 PCBs (congeners 28, 44, 49, 52, 66, 74, 87, 99, 101, 105, 110, 114, 118, 128, 138, 146, 149, 151, 153, 156, 157, 167, 170, 172, 177, 178, 180, 183, 187, 189, 194, 195, 196, 201, 206, and 209); and *e*) 7 PFCs [2-(*N*-ethyl-perfluorooctane sulfonamido) acetate (Et-PFOSA-AcOH), 2-(*N*-methyl-perfluorooctane sulfonamido) acetate (Me-PFOSA-AcOH), perfluorodecanoate (PFDeA), perfluorononanoate (PFNA), perfluorooctane sulfonamide (PFOSA), perfluorooctane sulfonate (PFOS), and perfluorooctanoate (PFOA)].

Serum concentrations were measured using isotope dilution high-resolution mass spectrometry for OCPs, PBBs, PBDEs, and PCBs, and isotope dilution tandem mass spectrometry for PFCs, following standard published operating procedures as described previously (Kato et al. 2011; Kuklenyik et al. 2005; Sjödin et al 2004). We did not perform automatic substitution of concentrations below the limit of detection or lipid adjustment in order to minimize bias associated with such practices when estimating health effects (Richardson and Ciampi 2003; Schisterman et al. 2005, 2006). Serum concentrations of cotinine [quantified using liquid chromatography-isotope dilution tandem mass spectrometry (Bernert et al. 1997)] and PFCs are reported in nanograms per milliliter; all other chemical concentrations are reported in nanograms per gram of serum. Serum lipids, quantified using commercially available enzymatic methods (Akins et al. 1989), were reported as total serum lipids (nanograms per gram of serum) using established methods based on individual components including phospholipids, triglycerides, total cholesterol, and free cholesterol (Phillips et al. 1989).

*Operational definitions*. Couple fecundity was measured by TTP, which denotes the number of menstrual cycles required by couples to achieve an hCG pregnancy. Couples achieving pregnancy within the first few weeks of enrollment or before a fully observed menstrual cycle were defined as having a TTP of 0; couples not achieving pregnancy after 12 months of trying were censored (TTP > 12). Definitions of relevant covariates included BMI (measured weight in kilograms divided by height in meters squared), gravidity (number of pregnancies), parity (number of live births), and smoking status based on serum cotinine concentration (continous).

*Statistical analysis*. In the descriptive phase of analysis, we assessed the distributions of all exposures and relevant covariates. We analyzed data under the missing-at-random assumption. Specifically, we implemented Markov Chain Monte Carlo methods to impute missing chemical, cotinine, and lipid (≤ 4%) data arising from insufficient blood for analysis (Schafer 1997). We used other chemical exposures for the imputation process. Geometric means (GMs) and 95% confidence intervals (CIs) were calculated for all chemicals, cotinine, and serum lipids.

We used daily journals supplemented with fertility monitors as needed to define menstrual cycles distinct from episodic bleeding. Specifically, a menstrual cycle denoted the interval (in days) from the onset of bleeding that increased in intensity and lasted ≥ 2 days to the onset of the next similar bleeding episode. Pregnancy was defined as a positive (hCG-confirmed) test on the day of expected menstruation.

The analytic phase was conducted in two parts. First, we estimated unadjusted fecundability odds ratios (FORs) and accompanying 95% CIs for all 63 chemicals by class (i.e., OCPs, PBB, PBDEs, PCBs, PFCs) using Cox models (Cox 1972) for discrete survival time (SAS version 9.2; SAS Institute Inc., Cary, NC) to estimate the odds of becoming pregnant during each cycle given exposure and conditional on not being pregnant in the previous cycle. This model allows the odds for pregnancy to vary from cycle to cycle through a cycle-varying intercept. Each chemical concentration (nanograms per gram of serum or wet weight) was log-transformed and divided by its standard deviation to rescale concentrations for biologic interpretation of the FORs, given the small unit size of chemical concentrations. Next, for chemicals that were significantly associated with TTP on the basis of unadjusted estimates, we ran additional models adjusted for *a priori* potential confounders [i.e., continuous age, BMI, serum cotinine, and serum lipids (except for PFC models); research site (Michigan or Texas); and the sum of the log-transformed serum concentrations of all other measured chemicals in the same class as the chemical being evaluated (American Society for Reproductive Medicine 2008a; Augood et al. 1998; Hassan and Killick 2004; Ramlau-Hansen et al. 2007)]. In addition, we accounted for left truncation reflecting time (≤ 2 months) couples did not use contraception before enrollment into the study. In this approach we assumed that months correspond to menstrual cycles and that all unobserved time is at risk for pregnancy despite the absence of data on sexual intercourse in relation to the fertile window. Underlying linearity for all continuous covariates were assessed using the Kolmogorov-type supremum test based on martingale residuals, and the proportional hazards assumptions were verified for all discrete-time models (Grambsch and Therneau 1994; Therneau and Grambsch 2000).

We also evaluated interactions between each chemical and age categorized as ≤ 27 versus > 27 years for females and ≤ 28 versus > 28 years for males based on previous evidence for this categorization (Dunson et al. 2002) and as corroborated in our cohort. However, we did not include interactions in our final models because none were observed.

Finally, we ran separate models adjusted for parity in sensitivity analyses, given the uncertain causal relationship between parity, POPs, and TTP. Specifically, we modeled parity conditional on gravidity by categorizing it as no prior pregnancy, prior pregnancy without live birth(s), or prior pregnancy with live birth(s) (Buck Louis et al. 2006).

Separate models were run for each chemical and partner. The concentrations of the chemicals evaluated were highly correlated with each other [for correlations in samples from females and males, see Supplemental Material, Figures S1 and S2, respectively (http://dx.doi.org/10.1289/ehp.1205301)] and between partners (see Supplemental Material, Figure S3; *r* = 0.71–0.97), which precluded joint modeling. Couples who withdrew from the study before pregnancy or before completing 12 months of follow-up (*n* = 100) or who were not pregnant after 12 months of follow-up (*n* = 54) were censored in all analyses. Statistical significance (*p* < 0.05) was determined using the chi-square statistic for categorical data, Student’s *t*-test or Wilcoxon nonparametric test for continuous data, or 95% CIs that excluded one. We did not adjust for multiple comparisons consistent with the exploratory nature of this work.

## Results

Sociodemographic and lifestyle characteristics differed between couples who became pregnant or completed 12 months of follow-up and those who withdrew before pregnancy or the end of follow-up (Table 1). Participants who withdrew were more likely to self-identify as nonwhite and to be without health insurance. Compared with women who completed the study, those who withdrew had significantly higher mean BMIs (27.2 vs. 29.4 kg/m^2^) and log-transformed serum cotinine concentrations (0.51 ng/mL vs. 1.08 ng/mL). Men who withdrew from the study had higher log-transformed serum cotinine concentrations (1.98 ng/mL) than those who completed the study (1.04 ng/mL). The probability of pregnancy at cycles 1, 3, 6, and 12 were 0.27 (95% CI: 0.23, 0.31), 0.52 (95% CI: 0.48, 0.57), 0.68 (95% CI: 0.64, 0.73), and 0.81 (95% CI: 0.76, 0.85), respectively.

**Table 1:** Comparison of study cohort by completion status, LIFE Study, 2005–2009.

Characteristic	Completed protocol (n = 401)	Withdrew (n = 100)
Females
Age	29.9 ± 3.9	30.4 ± 4.9
Gravidity	1.0 ± 1.2	1.3 ± 1.5
Parity	0.6 ± 0.8	0.7 ± 1.0
BMI (kg/m2)	27.2 ± 7.1**	29.4 ± 7.8
Serum lipids (ng/g)	617.1 ± 115.1	642.3 ± 157.2
Serum cotinine (ng/mL)a	0.51 ± 1.40#	1.08 ± 1.19
Nonwhite	74 (19)#	34 (34)
≤ High school graduate/GED	21 (5)	10 (10)
No health insurance	20 (5)#	20 (20)
Males
Age	31.7 ± 4.7	32.1 ± 5.8
BMI (kg/m2)	29.8 ± 5.5	29.7 ± 5.8
Serum lipids (ng/g)	728.9 ± 214.1	741.5 ± 220.9
Serum cotinine (ng/mL)a	1.04 ± 2.02##	1.98 ± 2.54
Nonwhite	73 (18)#	34 (34)
≤ High school graduate/GED	26 (7)#	23 (23)
No health insurance	28 (7)*	14 (14)
GED, General Educational Development (equivalent to high school diploma). Values are mean ± SE or n (%). aLog-transformed. *p < 0.05. **p < 0.01. #p < 0.001. ##p = 0.0001.		

Table 2 presents the GMs and 95% CIs for chemicals that were significantly associated with fecundity based on the unadjusted FORs; for corresponding estimates for all other chemicals see Supplemental Material, Tables S1 and S2 (http://dx.doi.org/10.1289/ehp.1205301). Among the chemicals shown in Table 2, GMs were comparable or higher in couples who withdrew before 12 months of follow-up or did not become pregnant during 12 months of follow-up compared with couples who became pregnant during follow up, although only values for PBDE 183 and PCB 138 in men were significantly different (0.003 vs. 0.002; *p* < 0.01 and 0.044 vs. 0.038; *p* < 0.05, respectively).

**Table 2:** GMs (95% CIs) of persistent environmental chemical concentrations by observed pregnancy status during follow-up, LIFE Study, 2005–2009.

Table 2.				
Chemical (ng/g serum)	LOD	Percent < LOD	Achieved pregnancy (n = 347)	Withdrew/not pregnant (n = 154)a
Females				
HCB	0.013	< 1	0.046 (0.045–0.048)	0.048 (0.044–0.051)
PCB 118b	0.0026	< 1	0.017 (0.016–0.018)	0.018 (0.016–0.020)
PCB 167b	0.0026	75	0.003 (0.003–0.003)	0.004 (0.003–0.004)
PCB 209	0.0026	77	0.002 (0.002–0.002)	0.002 (0.002–0.002)
PFOSA (ng/mL)	0.1	90	0.110 (0.100–0.122)	0.126 (0.106–0.151)
Males				
p,p’-DDE	0.013	< 1	0.766 (0.721–0.814)	0.818 (0.737–0.908)
PBDE 183	0.0026	67	0.002 (0.002–0.002)**	0.003 (0.002–0.003)
PCB 101	0.0026	62	0.003 (0.003–0.003)	0.003 (0.002–0.003)
PCB 138	0.0026	< 1	0.038 (0.036–0.041)*	0.044 (0.039–0.049)
PCB 153	0.0026	< 1	0.057 (0.053–0.061)	0.063 (0.057–0.071)
PCB 156b	0.0026	7	0.007 (0.007–0.008)	0.008 (0.007–0.009)
PCB 157b	0.0026	71	0.002 (0.002–0.003)	0.003 (0.002–0.003)
PCB 167b	0.0026	71	0.003 (0.003–0.003)	0.004 (0.003–0.004)
PCB 170	0.0026	1	0.017 (0.016–0.018)	0.019 (0.017–0.021)
PCB 172	0.0026	62	0.003 (0.003–0.003)	0.003 (0.003–0.004)
PCB 180	0.0026	< 1	0.044 (0.041–0.048)	0.049 (0.043–0.054)
PCB 209	0.0026	51	0.003 (0.003–0.003)	0.003 (0.003–0.003)
LOD, limit of detection of the analytical method. Chemicals shown here are those with a significant unadjusted fecundability odds ratio. For values of all other chemicals, see Supplemental Material, Tables S1 and S2 (http://dx.doi.org/10.1289/ehp.1205301). aIncludes couples who either withdrew from the study and those who did not become pregnant. bDioxin-like compounds. *p < 0.05. **p < 0.01.				

Of all the chemicals tested in models adjusting for left truncation or any time off contraception before enrollment, only PCB 101 in men had a significant positive association with TTP (unadjusted or adjusted), indicating a shorter time to pregnancy (FOR = 1.28; 95% CI: 1.09, 1.51) [Table 3; see also Supplemental Material, Tables S3 and S4 (http://dx.doi.org/10.1289/ehp.1205301)], whereas all remaining significant unadjusted FORs indicated a longer TTP (Table 3). The specific chemicals that were significantly associated with reduced FORs differed between females and males, with considerably more significant negative associations noted for exposures in men. Chemicals with significant negative associations based on unadjusted models were *p,p´*-DDE, PBDE 183, and PCB congeners 101, 138, 153, 156, 157, 167, 170, 172, 180, and 209 in males, and HCB, PFOSA, and PCB congeners 118, 167, and 209 in females. In adjusted models, females’ serum concentrations of PCBs 118, 167, and 209 were associated with an 18–21% reduction in fecundability per 1 SD increase in log-transformed serum concentrations; PFOSA was associated with an 18% reduction in fecundability per 1 SD increase (FOR = 0.82; 95% CI: 0.71, 0.95). When male serum concentrations were modeled with adjustment for covariates and left truncation, fecundability was reduced from 17% to 29% for *p,p´*-DDE and PCB congeners 138, 156, 157, 167, 170, 172, and 209.

**Table 3:** FORs (95% CIs) for environmental chemicals^*a*^ by partner and model, LIFE Study, 2005–2009.

Chemicalb	Unadjusted	Adjustedc	Adjustedd	Sensitivity modele
Females
HCB	0.87 (0.77, 1.00)*	0.95 (0.81, 1.11)	0.94 (0.80, 1.10)	0.96 (0.82, 1.13)
PCB 118f	0.87 (0.77, 0.99)	0.88 (0.75, 1.02)	0.82 (0.68, 0.98)	0.84 (0.70, 1.01)
PCB 167f	0.88 (0.78, 0.99)	0.87 (0.76, 1.00)	0.79 (0.64, 0.97)	0.81 (0.66, 1.00)*
PCB 209	0.87 (0.75, 1.00)*	0.86 (0.72, 1.02)	0.82 (0.68, 0.99)	0.77 (0.62, 0.95)
PFOSA (ng/mL)	0.82 (0.72, 0.93)	0.81 (0.70, 0.94)	0.82 (0.71, 0.95)	0.82 (0.71, 0.95)
Males
p,p’-DDE	0.88 (0.78, 0.99)	0.83 (0.70, 0.97)	0.83 (0.70, 0.97)	0.80 (0.67, 0.94)
PBDE 183	0.83 (0.71, 0.96)	0.86 (0.73, 1.01)	0.86 (0.73, 1.01)	0.85 (0.72, 1.00)*
PCB 101	1.18 (1.03, 1.36)	1.27 (1.08, 1.49)	1.28 (1.09, 1.51)	1.24 (1.05, 1.46)
PCB 138	0.84 (0.74, 0.97)	0.83 (0.70, 0.99)	0.71 (0.52, 0.98)	0.69 (0.50, 0.94)
PCB 153	0.86 (0.75, 0.98)	0.86 (0.73, 1.02)	0.67 (0.42, 1.06)	0.68 (0.43, 1.06)
PCB 156f	0.85 (0.75, 0.96)	0.84 (0.71, 0.99)	0.77 (0.62, 0.96)	0.76 (0.61, 0.94)
PCB 157f	0.86 (0.77, 0.97)	0.87 (0.75, 1.00)	0.83 (0.70, 0.97)	0.82 (0.70, 0.96)
PCB 167f	0.86 (0.75, 0.98)	0.85 (0.73, 0.99)	0.82 (0.70, 0.96)	0.82 (0.70, 0.96)
PCB 170	0.85 (0.75, 0.96)	0.84 (0.71, 1.00)*	0.74 (0.56, 0.98)	0.75 (0.58, 0.96)
PCB 172	0.87 (0.77, 0.99)	0.88 (0.75, 1.04)	0.82 (0.68, 0.99)	0.81 (0.67, 0.98)
PCB 180	0.87 (0.76, 0.98)	0.88 (0.74, 1.04)	0.81 (0.66, 1.00)	0.81 (0.66, 0.98)
PCB 209	0.84 (0.73, 0.96)	0.83 (0.70, 0.97)	0.78 (0.65, 0.94)	0.78 (0.65, 0.93)
aRestricted to chemicals with a significant association with fecundability based on unadjusted models; see Supplemental Material, Tables S3 and S4 (http://dx.doi.org/10.1289/ehp.1205301) for corresponding estimates for all other chemicals evaluated. bSerum concentrations were log-transformed then rescaled by their SDs to enhance the interpretation of effect sizes. cAdjusted for the sum of all other chemicals in the same chemical class, age (categorized), BMI (continuous), log-transformed serum cotinine (continuous), log-transformed serum lipids (continuous) except in PFC models, and site (Michigan or Texas). dAdjusted for left truncation to account for time off contraception before enrollment and sum of all other chemicals in the class of compounds, age (categorized), BMI (continuous), cotinine (continuous), lipids (continuous) except in PFC models, and site (Michigan/Texas). eSensitivity analysis adjusting for parity conditional on gravidity, in addition to left truncation and the other covariates listed above. fDioxin-like compounds. *95% CI significant before rounding to two decimal places (≤ 0.998).				

We observed a reduction in fecundability for PCBs reported to be dioxin-like (PCBs 118, 156, 157, and 167) compared with those without dioxin-like properties, although there were some differences between the sexes. PCB congeners 167 and 209 were consistently associated with reduced fecundability in females (FOR = 0.79; 95% CI: 0.64, 0.97 and FOR = 0.82; 95% CI: 0.68, 0.99, respectively) and males (FOR = 0.82; 95% CI: 0.70, 0.96 and FOR = 0.78; 95% CI: 0.65, 0.94, respectively). Adjusting for parity had little influence on model estimates [Table 3; see also Supplemental Material, Tables S3 and S4 (http://dx.doi.org/10.1289/ehp.1205301)].

## Discussion

Findings from the LIFE Study, a prospective cohort of couples enrolled prior to conception and followed for up to a year while attempting to become pregnant, provide empirical evidence that selected persistent environmental chemicals from various chemical classes (i.e., OCPs, PCBs, and PFCs) may adversely affect couple fecundability. Serum concentrations among the LIFE Study participants were largely below those reported for U.S. populations during a comparable time period (Centers for Disease Control and Prevention 2009), possibly reflecting the younger age structure of the LIFE Study cohort relative to the United States as a whole.

A novel finding is that the chemicals associated with reduced couple fecundability differed between males and females, but with a larger number of associations observed for males. We previously observed a similar finding for heavy metals and TTP in the LIFE Study, in which increased concentration in male partners was associated with a longer TTP (Buck Louis et al. 2012). These findings underscore the importance of males when assessing couple-dependent reproductive outcomes such as TTP. However, serum concentrations of mono-*ortho* PCB 167 and non–dioxin-like PCB 209 were associated with approximately a 20% reduction in the probability of an hCG-detected pregnancy per standard deviation increase in the log-transformed chemical concentration in both men and women, although it is important to note that serum concentrations of these PCB congeners were below the limit of detection in 71% and 77% of women and men, respectively, for PCB 167 and in 77% and 51% of women and men for PCB 209. Still, we know of no *a priori* reason or empirical evidence that supports a systematic difference in laboratory detection capability by couple fecundability, particularly given the blinding of laboratory personnel to fecundity status in the LIFE Study. The findings warrant further inquiry, given the study’s exploratory nature.

Interpreting our findings for partner-specific associations in the context of the previous literature is limited by the absence of similar preconception couple-based cohorts with exposure characterization for a mixture of persistent chemicals and 12 cycles of follow-up consistent with the clinical diagnosis of infertility (American Society for Reproductive Medicine 2008b). Previous studies have assessed selected chemicals and TTP, but these studies were primarily among pregnant women with blood collection at varying times during gestation and retrospectively reported TTP.

Several findings from the LIFE Study are globally consistent with earlier studies that reported reduced FORs for various PCBs (Axmon et al. 2005; Buck Louis et al. 2009; Gesink Law et al. 2005), although only the results reported by Axmon et al. (2005) were statistically significant. Our findings also corroborate the lack of an association between female concentrations of *p,p´-*DDT, *o,p´-*DDT, and *p,p´-*DDE and fecundability (Harley et al. 2008). Unlike the findings of Harley et al. (2010), we did not observe a relationship between PBDEs and FORs, possibly reflecting differences in the adjusted models or the earlier studies’ use of retrospectively measured TTP. Recently, three papers have addressed the relationship between select PFCs and fecundity (Fei et al. 2009; Vestergaard et al. 2012; Whitworth et al. 2012), although only one used a prospective couple-based cohort design (Vestergaard et al. 2012). Specifically, Vestergaard et al. (2012) observed no consistent pattern between eight PFCs, including PFOSA, measured in serum from 222 (52%) participating female partners who were followed for up to six cycles of attempting to become pregnant in 1992–1995. Important differences exist between this Danish cohort and the LIFE Study, including a shorter duration of follow-up (6 vs. 12 cycles) in the earlier study, and choice of model specification with regard to the handling of PFCs in the context of other POPs and potential confounders. In the LIFE Study, when we excluded 173 (8%) cycles without intercourse during the fertile window, average TTP (4.4 cycles) changed slightly (0.4 cycles). Our PFOSA finding remained even when we limited the analysis to six cycles of follow-up (FOR = 0.83; 95% CI: 0.70, 0.98). Two other studies of plasma PFOA and PFOS concentrations were restricted to women who had live births, and both relied upon retrospectively reported TTP, which was defined categorically (< 6, 6–12, > 12 months) (Fei et al. 2009) or dichotomously as subfecundity (TTP > 12 months) (Whitworth et al. 2012). Fei et al. (2009) reported a significant trend between PFOA and PFOS concentrations and FORs < 1 reflecting a longer TTP. Whitworth et al. (2012) reported higher odds of subfecundity, but only among parous women, a finding the authors interpreted as evidence of reverse causation, as first suggested by Olsen et al. (2009). The inclusion of parity conditional on gravidity in our sensitivity models did not substantially alter associations, which we believe is inconsistent with reverse causation. However, the association between serum PFOSA concentration in women and longer TTP, as indicated by the FOR < 1, must be interpreted with caution because concentrations were nondetectable in 90% of samples, possibly because U.S. production of PFOSA ceased in 2002.

The magnitude of negative associations with fecundability estimated for several of the POPs assessed in the LIFE Study are comparable to associations with other recognized fecundity determinants such as male and female age, BMI, and cigarette smoking (Bolumar et al. 1996; Dunson et al. 2002; Menken et al. 1986; Ramlau-Hansen et al. 2007) that we adjusted for in our statistical models. These findings underscore the importance of environmental factors that may affect couple fecundity as measured by TTP. Our findings are relatively consistent with studies of women undergoing assisted reproductive technologies that can examine associations with early reproductive outcomes (e.g., fertilization, cleavage, implantation) that are not observable in the general population. For example, evidence of negative associations between TTP and serum concentrations of HCB and PCB 118 in women is consistent with findings for implantation failures associated with these exposures among women undergoing assisted reproductive technologies (Mahalingaiah et al. 2012; Meeker et al. 2011). Although speculative, these findings suggest that associations between these chemicals and longer TTPs may reflect, in part, diminished implantation success. Still, our findings have important limitations, including the absence of information on the timing of exposures during sensitive windows for human reproduction (e.g., folliculogenesis, spermatogenesis), the absence of exposure data on short-lived chemicals such as bisphenol A and phthalates (Crain et al. 2008; Jurewicz and Hanke 2011), potential selection biases arising from enrollment of couples planning pregnancies, and possible residual confounding associated with more educated women using the monitor more effectively than lesser-educated women. We did not observe any differences in frequency or timing of intercourse as aided by the monitor and female education, nor did women experience difficulties complying with the monitor (data not shown).

The etiologic mechanisms by which endocrine-disrupting chemicals (EDCs), including those quantified in the LIFE Study, may affect human reproduction remain elusive, but globally these mechanisms are hypothesized to affect hormonal milieu through alterations in the production, release, transport, metabolism, and/or elimination of hormones (Sonnenschein and Soto 1998). With regard to ovarian function, experimental and human evidence suggests that EDCs alter both the expression/activity of enzymes required for ovarian sex steroid synthesis/catabolism, and the expression/ability of hormone receptors to bind endogenous ligands, as recently reviewed by Craig et al. (2011). Such changes that occur within the ovary are not in isolation because other endocrine organs that are relevant to reproduction, such as the thyroid, also may be affected by EDCs (Boas et al. 2012). The potential for diverse mechanisms of action underscore the need to consider couples’ exposures in relation to a broad spectrum of human reproductive outcomes, including altered hormonal profiles or sexual libido in either partner, semen quality in the male partner (Hauser et al. 2006), and changes in menstrual and ovarian cycles (Perry et al. 2006) and effects on ovulation or implantation in the female partner. Each of these end points, either alone or in combination, may manifest as a longer TPP.

Delineating an underlying causal model that might explain associations between EDCs and TTP remains a critical data gap. An exposome approach that captures the totality of nongenetic exposures from conception onward (Wild 2005) would allow chemical exposures to be evaluated in the context of lifestyle, behavior, and macro-level factors that also may be relevant to human fecundity and fertility. The need for a comprehensive approach to improve understanding of risk factors and underlying mechanisms has been proposed in relation to the testicular dysgenesis syndrome (Skakkebaek et al. 2001) and, subsequently, the ovarian dysgenesis syndrome (Buck Louis et al. 2011a), both of which may result in part from early exposures that may permanently reprogram fecundity and have implications across the life span. Such conceptual and methodologic approaches will facilitate understanding of the up- and down-stream effects that EDCs may pose for human reproduction and health.

## Supplemental Material

(254 KB) PDF
