# Supplementary material for: Persistent Environmental Pollutants and Couple Fecundity: The LIFE Study
Source: Environ Health Perspect. 2012 Nov 14;121(2):231–6. doi: 10.1289/ehp.1205301 (PMC3569685; doi:10.1289/ehp.1205301)

Supplemental Material

Persistent Enviromental Pollutants and Couple Fecundity: The LIFE Study

Germaine M. Buck Louis, Rajeshwari Sundaram, Enrique F. Schisterman, Anne M. Sweeney, Courtney D. Lynch, Robert E. Gore-Langton, Jose Maisog, Sungduk Kim, Zhen Chen and Dana B. Barr

Supplemental Materials - Table of Contents

Supplemental Table S1. Geometric mean comparison of females' persistent environmental chemical concentrations by observed pregnancy status during follow-up, LIFE Study, 2005-2009. See pages 2-4.

Supplemental Table S1. Geometric mean comparison of males' persistent environmental chemical concentrations by observed pregnancy status during follow-up, LIFE Study, 2005-2009. See pages 5-7.

Supplemental Table S3. Female partners' serum concentrations and fecundability odds ratios that did not achieve significance, LIFE Study, 2005-2009. See pages 8-11.

Supplemental Table S4. Male partners' serum concentrations and fecundability odds ratios that did not achieve significance, LIFE Study, 2005-2009. See pages 12-15.

Supplemental Figure S1. Cross-correlation matrix of chemicals in females against one another. See page 16.

Supplemental Figure S2. Cross-correlation matrix of chemicals in males against one another. See page 17.

Supplemental Figure S3. Cross-correlation matrix of chemicals in males against chemicals in females. See page 18.

Supplemental Table S1. Geometric mean comparison of females' persistent environmental chemical concentrations by observed pregnancy status during follow-up, LIFE Study, 2005-2009.

Chemical (ng/g serum)	% <LOD	Became Pregnant (n=347) Geometric mean (95% CI)	Withdrew or not Pregnant during Followup (n=154)[a] Geometric mean (95% CI)
**PBB:**			
153	12	0.008 (0.007-0.009)	0.008 (0.006-0.010)
**PBDEs:**			
17	81	0.001 (0.001-0.002)	0.002 (0.001-0.002)
28	24	0.010 (0.009-0.011)	0.011 (0.010-0.013)
47	< 1	0.119 (0.106-0.134)	0.133 (0.112-0.157)
66	85	0.001 (0.001-0.002)	0.001 (0.001-0.002)
85	58	0.003 (0.002-0.003)	0.003 (0.002-0.003)
99	24	0.021 (0.019-0.024)	0.024 (0.020-0.029)
100	<1	0.025 (0.022-0.028)	0.028 (0.024-0.034)
153	<1	0.046 (0.041-0.052)	0.044 (0.037-0.053)
154	59	0.003 (0.002-0.003)	0.003 (0.003-0.004)
183	82	0.002 (0.002-0.002)	0.002 (0.002-0.002)
**PCBs:**			
28	66	0.006 (0.005-0.006)	0.006 (0.006-0.007)
44	87	0.002 (0.002-0.002)	0.002 (0.002-0.002)
49	99	0.001 (0.000-0.001)	0.000 (0.000-0.001)
52	96	0.001 (0.001-0.001)	0.001 (0.001-0.001)
66	38	0.003 (0.003-0.003)	0.003 (0.003-0.004)
74	<1	0.014 (0.013-0.015)	0.014 (0.012-0.015)
87	93	0.001 (0.001-0.002)	0.002 (0.001-0.002)
99	1	0.010 (0.010-0.011)	0.011 (0.010-0.012)
101	72	0.002 (0.002-0.002)	0.002 (0.002-0.003)
105	26	0.004 (0.004-0.004)	0.004 (0.004-0.005)
110	89	0.001 (0.001-0.002)	0.001 (0.001-0.002)

Chemical (ng/g serum)	% <LOD	Became Pregnant (n=347)<br><br>Geometric mean (95% CI)	Withdrew or not Pregnant during Followup (n=154)[a]<br>Geometric mean (95% CI)
**PCBs:**			
118[b]	<1	0.017 (0.016-0.018)	0.018 (0.016-0.020)
128	98	0.003 (0.002-0.004)	0.003 (0.002-0.004)
138	<1	0.031 (0.029-0.034)	0.033 (0.029-0.037)
146	20	0.006 (0.005-0.006)	0.006 (0.005-0.007)
149	97	0.001 (0.001-0.001)	0.001 (0.001-0.002)
151	98	0.001 (0.001-0.002)	0.002 (0.001-0.003)
153	<1	0.044 (0.041-0.047)	0.044 (0.039-0.050)
156	16	0.006 (0.006-0.006)	0.006 (0.005-0.007)
157[b]	78	0.002 (0.002-0.002)	0.002 (0.002-0.003)
167[b]	75	0.003 (0.003-0.003)	0.004 (0.003-0.004)
170	2	0.012 (0.012-0.013)	0.012 (0.011-0.014)
172	80	0.002 (0.002-0.003)	0.003 (0.002-0.003)
177	58	0.003 (0.003-0.003)	0.003 (0.003-0.003)
178	64	0.003 (0.003-0.004)	0.003 (0.003-0.004)
180	<1	0.031 (0.029-0.033)	0.029 (0.026-0.033)
183	31	0.005 (0.004-0.005)	0.005 (0.004-0.005)
187	7	0.011 (0.010-0.011)	0.011 (0.010-0.013)
189[b]	98	0.001 (0.001-0.001)	0.001 (0.001-0.002)
194	19	0.008 (0.007-0.008)	0.007 (0.007-0.008)
195	77	0.003 (0.002-0.003)	0.003 (0.002-0.003)
196	8	0.007 (0.007-0.008)	0.007 (0.007-0.008)
201	14	0.007 (0.006-0.007)	0.006 (0.006-0.007)
206	32	0.004 (0.003-0.004)	0.004 (0.003-0.004)
209	77	0.002 (0.002-0.002)	0.002 (0.002-0.002)
**PFCs (ng/mL):**			
Et-PFOSA-AcOH	97	0.114 (0.102-0.127)	0.116 (0.102-0.132)
Me-PFOSA-AcOH	25	0.288 (0.264-0.315)	0.332 (0.288-0.384)
PFDeA	10	0.385 (0.359-0.413)	0.349 (0.316-0.386)

**Chemical (ng/g serum)**	**% <LOD**	**Became Pregnant (n=347)<br><br>Geometric mean (95% CI)**	**Withdrew or not Pregnant during Followup (n=154)[a]<br>Geometric mean (95% CI)**
PFNA	1	1.176 (1.103-1.254)	1.112 (1.015-1.217)
**PFCs (ng/mL):**			
PFOA	< 1	3.112 (2.908-3.331)	3.101 (2.849-3.376)
PFOS	< 1	11.764 (11.012-12.566)	11.088 (9.947-12.359)
PFOSA	90	0.110 (0.100-0.122)	0.126 (0.106-0.151)
**OCPs:**			
β-hexachlorocyclohexane	59	0.016 (0.014-0.018)	0.019 (0.016-0.024)
γ-hexachlorocyclohexane	99	0.005 (0.004-0.006)	0.006 (0.004-0.008)
Hexachlorobenzene	< 1	0.046 (0.045-0.048)	0.048 (0.044-0.051)
Mirex	78	0.007 (0.007-0.008)	0.008 (0.006-0.009)
o,p'-DDT	99	0.002 (0.002-0.003)	0.003 (0.002-0.003)
Oxychlordane	16	0.034 (0.032-0.036)	0.034 (0.030-0.037)
p,p'-DDE	< 1	0.590 (0.552-0.631)	0.661 (0.590-0.741)
p,p'-DDT	58	0.012 (0.011-0.013)	0.014 (0.012-0.015)
Trans-nonachlor	3	0.050 (0.046-0.054)	0.051 (0.045-0.057)

NOTE: None of the above differences achieved significance.

[a]Includes women who either withdrew from study at varying stages of trying and who did not become pregnant.

[b]Dioxin-like PCB congeners

Supplemental Table S2. Geometric mean comparison of males' persistent environmental chemical concentrations by observed pregnancy status during follow-up, LIFE Study, 2005-2009.

Chemical (ng/g serum)	% <LOD	Became Pregnant (n=347) Geometric mean (95% CI)	Withdrew or not Pregnant during Followup (n=154)[a] Geometric mean (95% CI)
**PBB:**			
153	6	0.012 (0.011-0.013)	0.014 (0.011-0.017)
**PBDEs:**			
17	80	0.001 (0.001-0.002)	0.002 (0.001-0.002)
28	28	0.010 (0.009-0.011)	0.011 (0.009-0.012)
47	1	0.119 (0.106-0.133)	0.135 (0.114-0.159)
66	56	0.003 (0.002-0.003)	0.003 (0.002-0.003)
85	24	0.023 (0.020-0.026)	0.027 (0.023-0.031)
99*	84	0.002 (0.001-0.002)	0.002 (0.001-0.002)
100	1	0.026 (0.023-0.029)	0.031 (0.026-0.037)
153	<1	0.068 (0.059-0.078)	0.068 (0.056-0.082)
154	57	0.003 (0.003-0.003)	0.003 (0.003-0.004)
183**	67	0.002 (0.002-0.002)	0.003 (0.002-0.003)
**PCBs:**			
28	73	0.005 (0.005-0.006)	0.005 (0.005-0.006)
44	85	0.002 (0.002-0.002)	0.002 (0.002-0.002)
49	99	0.001 (0.001-0.001)	0.000 (0.000-0.001)
52	94	0.001 (0.001-0.001)	0.001 (0.001-0.001)
66	49	0.003 (0.003-0.003)	0.003 (0.002-0.003)
74	1	0.014 (0.013-0.015)	0.015 (0.014-0.016)
87	91	0.002 (0.002-0.002)	0.002 (0.002-0.002)
99*	2	0.012 (0.011-0.013)	0.013 (0.012-0.015)
101	62	0.003 (0.003-0.003)	0.003 (0.002-0.003)
105	28	0.004 (0.004-0.004)	0.004 (0.004-0.005)
110	88	0.001 (0.001-0.002)	0.002 (0.001-0.002)

**Chemical (ng/g serum)**	**% <LOD**	**Became Pregnant (n=347) Geometric mean (95% CI)**	**Withdrew or not Pregnant during Followup (n=154)[a] Geometric mean (95% CI)**
**PCBs:**			
114	88	0.002 (0.001-0.002)	0.002 (0.001-0.002)
118[b]	1	0.018 (0.017-0.019)	0.019 (0.017-0.021)
128	96	0.002 (0.002-0.003)	0.003 (0.002-0.004)
138*	<1	0.038 (0.036-0.041)	0.044 (0.039-0.049)
146	10	0.007 (0.007-0.008)	0.008 (0.007-0.009)
149	96	0.001 (0.001-0.002)	0.001 (0.001-0.002)
151	96	0.002 (0.001-0.003)	0.002 (0.001-0.003)
153	<1	0.057 (0.053-0.061)	0.063 (0.057-0.071)
156[b]	7	0.007 (0.007-0.008)	0.008 (0.007-0.009)
157[b]	71	0.002 (0.002-0.003)	0.003 (0.002-0.003)
167[b]	71	0.003 (0.003-0.003)	0.004 (0.003-0.004)
170	1	0.017 (0.016-0.018)	0.019 (0.017-0.021)
172	62	0.003 (0.003-0.003)	0.003 (0.003-0.004)
177	40	0.004 (0.003-0.004)	0.004 (0.003-0.004)
178	42	0.004 (0.004-0.005)	0.004 (0.004-0.005)
180	<1	0.044 (0.041-0.048)	0.049 (0.043-0.054)
183	17	0.006 (0.005-0.006)	0.006 (0.006-0.007)
187	4	0.015 (0.014-0.016)	0.016 (0.014-0.018)
189[b]	93	0.002 (0.001-0.002)	0.002 (0.001-0.002)
194	10	0.011 (0.010-0.012)	0.012 (0.011-0.014)
195	62	0.003 (0.003-0.004)	0.003 (0.003-0.004)
196*	3	0.011 (0.010-0.012)	0.012 (0.010-0.013)
201	6	0.010 (0.009-0.011)	0.011 (0.010-0.012)
206	11	0.006 (0.005-0.006)	0.006 (0.005-0.007)
209	51	0.003 (0.003-0.003)	0.003 (0.003-0.003)
**PFCs (ng/mL):**			
Et-PFOSA-AcOH	97	0.111 (0.101-0.121)	0.131 (0.105-0.162)
Me-PFOSA-AcOH	22	0.310 (0.284-0.338)	0.342 (0.296-0.395)

Chemical	% <LOD	Became Pregnant (n=347)<br><br>Geometric mean (95% CI)	Withdrew or not Pregnant during Followup (n=154)[a]<br>Geometric mean (95% CI)
PFDeA	5	0.448 (0.422-0.476)	0.416 (0.381-0.454)
**PFCs (ng/mL):**			
PFNA	1	1.558 (1.472-1.649)	1.422 (1.296-1.561)
PFOA	<1	5.016 (4.777-5.268)	4.749 (4.400-5.125)
PFOS	<1	20.867 (19.574-22.244)	19.765 (17.994-21.710)
PFOSA	84	0.112 (0.103-0.121)	0.129 (0.108-0.155)
**OCPs:**			
β-hexachlorocyclohexane	55	0.017 (0.015-0.019)	0.018 (0.015-0.021)
γ-hexachlorocyclohexane	100	0.006 (0.005-0.008)	0.006 (0.004-0.007)
Hexachlorobenzene	<1	0.055 (0.053-0.057)	0.058 (0.055-0.062)
Mirex	55	0.013 (0.011-0.014)	0.013 (0.011-0.015)
o,p'-DDT	99	0.003 (0.003-0.003)	0.003 (0.003-0.003)
Oxychlordane	10	0.042 (0.039-0.045)	0.044 (0.040-0.049)
p,p'-DDE	<1	0.766 (0.721-0.814)	0.818 (0.737-0.908)
p,p'-DDT	43	0.014 (0.013-0.015)	0.015 (0.014-0.017)
Trans-nonachlor	2	0.067 (0.062-0.072)	0.071 (0.063-0.080)

*p < 0.05; **p < 0.01

[a]Includes women who either withdrew from study at varying stages of trying and who did not become pregnant.

[b]Dioxin-like PCB congeners

Supplemental Table S3. Female partners' serum concentrations and fecundability odds ratios that did not achieve significance, LIFE Study, 2005-2009.

Chemical (ng/g serum)	Standard Deviation	Unadjusted FOR (95% CI)	Adjusted[a] FOR (95% CI)	Adjusted[b] FOR (95% CI)	Sensitivity Adjusted Model[c] FOR (95% CI)
**POPs:**					
Mirex	0.0315	0.98 (0.85,1.13)	1.03 (0.90,1.18)	1.06 (0.91,1.23)	1.07 (0.92,1.24)
β-HCH	0.1346	0.95 (0.82,1.11)	1.04 (0.85,1.27)	1.04 (0.85,1.27)	1.02 (0.82,1.26)
γ-HCH	0.0022	1.02 (0.91,1.13)	1.00 (0.90,1.11)	1.01 (0.90,1.12)	1.03 (0.92,1.14)
o,p'-DDT	0.0046	0.94 (0.79,1.12)	1.08 (0.87,1.35)	1.09 (0.88,1.36)	1.06 (0.85,1.33)
Oxychlordane	0.0278	0.95 (0.84,1.07)	1.05 (0.92,1.20)	1.04 (0.91,1.19)	1.07 (0.93,1.22)
p,p'-DDE	0.3220	0.89 (0.78,1.01)	0.85 (0.71,1.01)	0.84 (0.70,1.00)	0.87 (0.73,1.04)
p,p'-DDT	0.0278	0.89 (0.74,1.06)	1.00 (0.75,1.32)	1.01 (0.76,1.34)	0.96 (0.72,1.28)
Trans-nonachlor	0.0527	0.94 (0.83,1.06)	1.02 (0.89,1.15)	1.02 (0.89,1.15)	1.03 (0.90,1.17)
**PBB:**					
153	0.0315	0.89 (0.75,1.05)	0.92 (0.79,1.08)	0.92 (0.79,1.08)	0.94 (0.80,1.10)
**PBDEs:**					
17	0.0039	0.95 (0.84,1.08)	0.95 (0.80,1.13)	0.95 (0.79,1.13)	0.94 (0.79,1.13)
28	0.0219	0.98 (0.88,1.09)	1.11 (0.85,1.44)	1.08 (0.83,1.42)	1.09 (0.83,1.44)
47	0.2340	0.94 (0.83,1.06)	0.88 (0.70,1.09)	0.87 (0.69,1.08)	0.84 (0.67,1.06)
66	0.0049	1.00 (0.90,1.11)	1.03 (0.88,1.21)	1.03 (0.88,1.20)	1.03 (0.88,1.21)
85	0.0135	0.98 (0.88,1.10)	1.12 (0.84,1.48)	1.09 (0.83,1.45)	1.11 (0.83,1.49)
99	0.0987	0.97 (0.86,1.09)	1.03 (0.78,1.36)	1.03 (0.78,1.37)	1.02 (0.76,1.37)
100	0.0871	0.96 (0.86,1.08)	1.01 (0.71,1.45)	1.01 (0.71,1.44)	1.02 (0.70,1.48)
153	0.1199	1.01 (0.90,1.14)	1.04 (0.91,1.19)	1.05 (0.92,1.20)	1.05 (0.92,1.21)
154	0.0123	0.96 (0.85,1.08)	0.98 (0.69,1.39)	0.96 (0.67,1.37)	0.96 (0.67,1.38)
183	0.0058	0.84 (0.64,1.11)	0.88 (0.69,1.12)	0.89 (0.70,1.13)	0.87 (0.65,1.15)

Chemical (ng/g serum)	Standard Deviation	Unadjusted FOR (95% CI)	Adjusted[a] FOR (95% CI)	Adjusted[b] FOR (95% CI)	Sensitivity Adjusted Model[c] FOR (95% CI)
**PCBs:**					
28	0.0830	1.08 (0.95,1.23)	1.09 (0.94,1.25)	1.32 (0.75,2.30)	1.34 (0.72,2.48)
44	0.0108	1.09 (0.96,1.24)	1.10 (0.88,1.38)	1.27 (0.85,1.90)	1.28 (0.80,2.07)
49	0.0042	1.09 (0.96,1.24)	1.12 (0.89,1.40)	1.30 (0.86,1.96)	1.29 (0.84,1.98)
52	0.0105	1.08 (0.95,1.23)	1.09 (0.87,1.37)	1.25 (0.85,1.85)	1.27 (0.80,2.02)
66	0.0225	1.07 (0.94,1.22)	1.09 (0.86,1.38)	1.23 (0.87,1.75)	1.22 (0.82,1.80)
74	0.0210	1.02 (0.88,1.18)	1.15 (0.82,1.60)	1.21 (0.86,1.70)	1.34 (0.92,1.95)
87	0.0012	0.95 (0.83,1.08)	0.97 (0.83,1.13)	0.97 (0.83,1.13)	0.98 (0.83,1.15)
99	0.0100	0.89 (0.78,1.01)	0.87 (0.73,1.04)	0.82 (0.68,1.00)	0.85 (0.70,1.03)
101	0.0034	1.06 (0.92,1.21)	1.06 (0.88,1.28)	1.06 (0.89,1.28)	1.04 (0.86,1.26)
105	0.0042	0.89 (0.78,1.01)	0.90 (0.77,1.05)	0.85 (0.71,1.01)	0.87 (0.73,1.04)
110	0.0017	1.03 (0.90,1.17)	1.02 (0.88,1.19)	1.03 (0.89,1.19)	1.03 (0.89,1.20)
114	0.0014	0.96 (0.85,1.09)	1.00 (0.86,1.16)	0.97 (0.83,1.13)	0.98 (0.83,1.15)
128	0.0008	0.95 (0.82,1.09)	0.97 (0.84,1.12)	0.96 (0.83,1.11)	0.96 (0.83,1.11)
138	0.0298	0.89 (0.79,1.01)	0.91 (0.77,1.06)	0.76 (0.53,1.10)	0.82 (0.59,1.14)
146	0.0059	0.92 (0.81,1.04)	0.92 (0.79,1.07)	0.84 (0.66,1.06)	0.84 (0.64,1.09)
149	0.0032	1.10 (0.93,1.30)	1.12 (0.94,1.33)	1.11 (0.93,1.32)	1.09 (0.91,1.30)
151	0.0040	1.15 (0.97,1.38)	1.17 (0.97,1.40)	1.15 (0.96,1.38)	1.11 (0.93,1.33)
153	0.0413	0.93 (0.82,1.05)	0.94 (0.81,1.09)	0.82 (0.56,1.20)	0.84 (0.57,1.23)
156[d]	0.0062	0.99 (0.88,1.11)	1.04 (0.89,1.21)	1.00 (0.85,1.18)	1.03 (0.87,1.22)
157[d]	0.0019	0.96 (0.85,1.08)	0.98 (0.85,1.13)	0.94 (0.81,1.10)	0.97 (0.84,1.13)
170	0.0124	0.95 (0.83,1.07)	0.97 (0.84,1.12)	0.90 (0.75,1.08)	0.91 (0.75,1.10)
172	0.0020	0.94 (0.83,1.07)	0.91 (0.77,1.06)	0.92 (0.78,1.07)	0.92 (0.78,1.08)
177	0.0041	1.07 (0.92,1.24)	0.92 (0.79,1.07)	1.14 (0.93,1.40)	1.14 (0.93,1.40)
178	0.0031	0.97 (0.85,1.11)	1.12 (0.94,1.33)	0.95 (0.80,1.13)	0.94 (0.79,1.12)
180	0.0293	0.96 (0.85,1.09)	1.17 (0.97,1.40)	0.93 (0.78,1.10)	0.93 (0.78,1.11)
183	0.0055	0.94 (0.81,1.10)	0.94 (0.81,1.09)	0.95 (0.77,1.16)	0.96 (0.78,1.17)
187	0.0130	0.97 (0.84,1.11)	1.04 (0.89,1.21)	0.94 (0.77,1.13)	0.93 (0.76,1.12)
189[d]	0.0008	0.98 (0.87,1.10)	0.98 (0.85,1.13)	0.95 (0.83,1.08)	0.95 (0.83,1.08)

Chemical (ng/g serum)	Standard Deviation	Unadjusted FOR (95% CI)	Adjusted[a] FOR (95% CI)	Adjusted[b] FOR (95% CI)	Sensitivity Adjusted Model[c] FOR (95% CI)
**PCBs:**					
194	0.0067	1.00 (0.89,1.14)	0.97 (0.84,1.12)	1.02 (0.87,1.20)	0.99 (0.84,1.16)
195	0.0020	0.95 (0.84,1.08)	0.91 (0.77,1.06)	0.94 (0.80,1.09)	0.94 (0.81,1.10)
196	0.0068	0.96 (0.84,1.09)	0.92 (0.79,1.07)	0.97 (0.81,1.16)	0.94 (0.78,1.13)
201	0.0071	1.00 (0.88,1.13)	1.12 (0.94,1.33)	1.00 (0.85,1.17)	0.96 (0.82,1.13)
206	0.0035	0.94 (0.82,1.07)	1.17 (0.97,1.40)	0.97 (0.83,1.14)	0.93 (0.80,1.10)
**PFCs (ng/mL):**					
Et-PFOSA-AcOH	0.0973	0.92 (0.79,1.07)	0.94 (0.82,1.08)	0.94 (0.82,1.09)	0.94 (0.81,1.08)
Me-PFOSA-AcOH	0.2549	0.92 (0.81,1.04)	0.95 (0.83,1.09)	0.96 (0.84,1.09)	0.93 (0.81,1.06)
PFDeA	0.2006	1.06 (0.95,1.19)	1.11 (0.95,1.29)	1.10 (0.95,1.28)	1.07 (0.92,1.25)
PFNA	0.3204	1.00 (0.89,1.13)	1.00 (0.84,1.19)	1.01 (0.85,1.20)	0.99 (0.84,1.18)
PFOS	0.5658	0.98 (0.87,1.11)	0.99 (0.85,1.17)	0.99 (0.85,1.17)	0.98 (0.83,1.15)
PFOA	0.4314	0.97 (0.86,1.09)	0.95 (0.82,1.11)	0.94 (0.81,1.10)	1.05 (0.89,1.23)

NOTE: Chemical serum concentrations were log transformed then rescaled by their standard deviations before analysis to enhance the interpretation of effect sizes. Cotinine and lipids concentrations were log transformed for analysis.

[a]Adjusted for individual chemical, sum of all remaining chemicals in the same chemical class, age (categorized), BMI (continuous), serum cotinine (continuous), serum lipids (continuous, not included in PFC models), and site (Michigan/Texas).

[b]Sensitivity model with left truncation to account for time off contraception before enrollment, and adjusting for individual chemical,

sum of all other chemicals in the class of compounds, age (categorized), BMI (continuous), serum cotinine (continuous), serum lipids (continuous, not included in PFC models), and site (Michigan/Texas).

[c]Sensitivity model with left truncation to account for time off contraception before enrollment, and adjusting for individual chemical, sum of all other chemicals in the class of compounds, parity conditional on gravidity, age (categorized), BMI (continuous), serum cotinine (continuous), serum lipids (continuous, not included in PFC models), and site (Michigan/Texas).

[d]Dioxin-like PCB congeners

Supplemental Table S4. Male partners' serum concentration of environmental chemicals and fecundability odds ratios that did not achieve significance, LIFE Study, 2005-2009.

Chemical (ng/g serum)	Standard Deviation	Unadjusted FOR (95% CI)	Adjusted[a] FOR (95% CI)	Adjusted[b] FOR (95% CI)	Sensitivity Adjusted Model[c] FOR (95% CI)
**POPs:**					
HCB	0.0266	0.90 (0.80,1.01)	0.92 (0.81,1.05)	0.94 (0.81,1.09)	0.92 (0.79,1.06)
Mirex	0.0704	1.00 (0.90,1.12)	0.97 (0.86,1.10)	1.05 (0.94,1.17)	1.05 (0.94,1.17)
β-HCH	0.1348	1.03 (0.91,1.15)	1.08 (0.93,1.26)	1.14 (0.98,1.33)	1.18 (1.00,1.38)
γ-HCH	0.0030	1.00 (0.90,1.12)	1.09 (0.90,1.32)	0.99 (0.89,1.11)	1.01 (0.90,1.13)
o,p'-DDT	0.0028	1.01 (0.89,1.14)	0.89 (0.78,1.02)	1.15 (0.97,1.36)	1.14 (0.96,1.34)
Oxychlordane	0.0388	0.95 (0.84,1.06)	0.96 (0.80,1.15)	1.06 (0.91,1.22)	1.02 (0.88,1.18)
p,p'-DDT	0.0301	0.98 (0.87,1.11)	1.10 (0.93,1.31)	1.11 (0.93,1.31)	1.08 (0.91,1.29)
Trans-nonachlor	0.0805	0.95 (0.84,1.07)	1.03 (0.89,1.18)	1.03 (0.89,1.18)	0.99 (0.86,1.15)
**PBB:**					
153	0.0683	0.91 (0.80,1.04)	0.98 (0.86,1.12)	0.98 (0.85,1.11)	0.96 (0.84,1.10)
**PBDEs:**					
17	0.0032	0.99 (0.88,1.12)	1.00 (0.86,1.16)	1.01 (0.86,1.17)	0.98 (0.84,1.14)
28	0.0161	0.98 (0.87,1.09)	0.92 (0.75,1.12)	0.92 (0.76,1.13)	0.92 (0.76,1.13)
47	0.2084	0.98 (0.88,1.10)	0.95 (0.83,1.10)	0.96 (0.83,1.11)	0.95 (0.82,1.09)
66	0.0029	1.07 (0.95,1.20)	1.11 (0.95,1.29)	1.11 (0.95,1.29)	1.11 (0.96,1.29)
85	0.0083	1.01 (0.90,1.14)	1.04 (0.83,1.30)	1.05 (0.84,1.31)	1.06 (0.85,1.32)
99	0.0752	1.00 (0.89,1.13)	1.02 (0.84,1.23)	1.03 (0.85,1.24)	1.02 (0.85,1.23)
100	0.0811	0.98 (0.87,1.10)	0.89 (0.67,1.19)	0.90 (0.68,1.20)	0.91 (0.68,1.22)
153	0.1919	1.01 (0.90,1.13)	1.02 (0.90,1.15)	1.01 (0.90,1.14)	1.02 (0.91,1.15)
154	0.0088	1.02 (0.90,1.15)	1.10 (0.88,1.37)	1.11 (0.89,1.38)	1.11 (0.90,1.38)
**PCBs:**					
28	0.0989	1.09 (0.96,1.24)	1.11 (0.96,1.27)	1.46 (0.67,3.19)	1.49 (0.65,3.41)

**Chemical (ng/g serum)**	**Standard Deviation**	**Unadjusted FOR (95% CI)**	**Adjusted[a] FOR (95% CI)**	**Adjusted[b] FOR (95% CI)**	**Sensitivity Adjusted Model[c] FOR (95% CI)**
**PCBs:**					
44	0.0103	1.07 (0.94,1.22)	1.13 (0.93,1.39)	1.23 (0.96,1.58)	1.24 (0.98,1.58)
49	0.0051	1.09 (0.96,1.24)	1.17 (0.95,1.44)	1.32 (0.93,1.89)	1.34 (0.98,1.84)
52	0.0130	1.10 (0.97,1.25)	1.20 (0.97,1.49)	1.51 (0.87,2.63)	1.47 (0.93,2.32)
66	0.0235	1.07 (0.94,1.22)	1.19 (0.95,1.50)	1.36 (0.95,1.94)	1.37 (0.98,1.92)
74	0.0259	1.00 (0.86,1.16)	1.13 (0.88,1.45)	1.20 (0.93,1.55)	1.18 (0.92,1.53)
87	0.0016	1.02 (0.89,1.16)	1.04 (0.90,1.19)	1.05 (0.92,1.21)	1.02 (0.89,1.17)
99	0.0156	0.90 (0.78,1.03)	0.88 (0.73,1.06)	0.85 (0.69,1.04)	0.82 (0.66,1.01)
105	0.0063	0.95 (0.84,1.08)	0.95 (0.81,1.11)	0.93 (0.79,1.11)	0.91 (0.76,1.08)
110	0.0020	1.01 (0.89,1.15)	1.02 (0.88,1.17)	1.01 (0.88,1.17)	0.96 (0.83,1.11)
114	0.0015	0.89 (0.79,1.02)	0.95 (0.82,1.10)	0.95 (0.82,1.11)	0.95 (0.82,1.10)
118[d]	0.0236	0.94 (0.83,1.07)	0.94 (0.81,1.10)	0.92 (0.78,1.09)	0.90 (0.75,1.06)
128	0.0012	0.99 (0.88,1.12)	1.01 (0.87,1.17)	0.99 (0.85,1.16)	0.99 (0.85,1.16)
146	0.0101	0.89 (0.77,1.02)	0.89 (0.75,1.04)	0.82 (0.66,1.02)	0.81 (0.66,0.99)
149	0.0045	1.13 (0.96,1.33)	1.18 (0.97,1.44)	1.17 (0.96,1.43)	1.17 (0.95,1.43)
151	0.0062	1.09 (0.92,1.28)	1.12 (0.94,1.33)	1.09 (0.92,1.30)	1.09 (0.91,1.31)
177	0.0067	0.98 (0.83,1.15)	1.09 (0.87,1.36)	1.06 (0.82,1.36)	1.06 (0.81,1.38)
178	0.0051	0.93 (0.82,1.05)	0.97 (0.82,1.16)	0.92 (0.77,1.11)	0.92 (0.76,1.11)
183	0.0083	0.92 (0.79,1.07)	0.98 (0.79,1.20)	0.91 (0.73,1.14)	0.91 (0.72,1.15)
187	0.0224	0.91 (0.79,1.04)	0.94 (0.79,1.11)	0.88 (0.73,1.06)	0.87 (0.72,1.06)
189	0.0011	0.90 (0.80,1.02)	0.93 (0.81,1.07)	0.91 (0.79,1.05)	0.91 (0.78,1.05)
194	0.0139	0.90 (0.79,1.02)	0.95 (0.80,1.12)	0.92 (0.77,1.10)	0.92 (0.77,1.10)
195	0.0035	0.92 (0.81,1.04)	0.97 (0.82,1.14)	0.94 (0.79,1.11)	0.92 (0.78,1.09)
196	0.0140	0.93 (0.82,1.06)	1.03 (0.86,1.22)	1.01 (0.83,1.22)	0.98 (0.81,1.20)
201	0.0169	0.96 (0.84,1.09)	1.04 (0.89,1.22)	1.04 (0.88,1.24)	1.03 (0.86,1.24)
206	0.0083	0.94 (0.83,1.07)	1.02 (0.88,1.19)	1.03 (0.86,1.22)	1.01 (0.85,1.21)
**PFCs (ng/mL):**					

Chemical (ng/g serum)	Standard Deviation	Unadjusted FOR (95% CI)	Adjusted[a] FOR (95% CI)	Adjusted[b] FOR (95% CI)	Sensitivity Adjusted Model[c] FOR (95% CI)
Et-PFOSA-AcOH	0.1012	0.92 (0.81,1.05)	0.92 (0.81,1.05)	0.93 (0.81,1.06)	0.91 (0.72,1.15)
Me-PFOSA-AcOH	0.2593	0.94 (0.84,1.06)	0.97 (0.86,1.10)	0.98 (0.87,1.12)	0.87 (0.72,1.06)
PFDeA	0.1901	1.08 (0.96,1.20)	1.08 (0.93,1.26)	1.08 (0.92,1.26)	0.91 (0.78,1.05)
PFNA	0.3177	1.08 (0.96,1.21)	1.09 (0.90,1.32)	1.10 (0.91,1.33)	0.92 (0.77,1.10)
PFOSA	0.0480	0.89 (0.79,1.01)	0.89 (0.78,1.02)	0.90 (0.79,1.02)	0.92 (0.78,1.09)
PFOS	0.5608	0.99 (0.87,1.12)	0.96 (0.80,1.15)	0.96 (0.80,1.15)	0.98 (0.81,1.20)
PFOA	0.3899	1.02 (0.90,1.15)	1.01 (0.88,1.17)	1.02 (0.89,1.18)	1.03 (0.86,1.24)

NOTE: Chemical serum concentrations were log transformed then rescaled by their standard deviations to enhance the interpretation of effect sizes. Cotinine and lipids concentrations were log transformed for analysis.

[*]CI significant before rounding to two decimal places (≤0.998).

[a]Adjusted for individual chemical, sum of all remaining chemicals in the same chemical class, age (categorized), BMI (continuous), serum cotinine (continuous), serum lipids (continuous, except in PFC models), and site (Michigan/Texas).

[b]Sensitivity model with left truncation to account for time off contraception before enrollment, and adjusting for individual chemical, sum of all remaining chemicals in the class of compounds, age (categorized), BMI (continuous), serum cotinine (continuous), serum lipids (continuous, except in PFC models), and site (Michigan/Texas).

[c]Sensitivity model with left truncation to account for time off contraception before enrollment, and adjusting for individual chemical, sum of all remaining chemicals in the class of compounds, parity conditional on gravidity, age (categorized), BMI (continuous), serum cotinine (continuous), serum lipids (continuous, except in PFC models), and site (Michigan/Texas).

[d]Dioxin-like PCB congeners

Figure S1. Cross−correlation matrix of chemicals in females against one another.

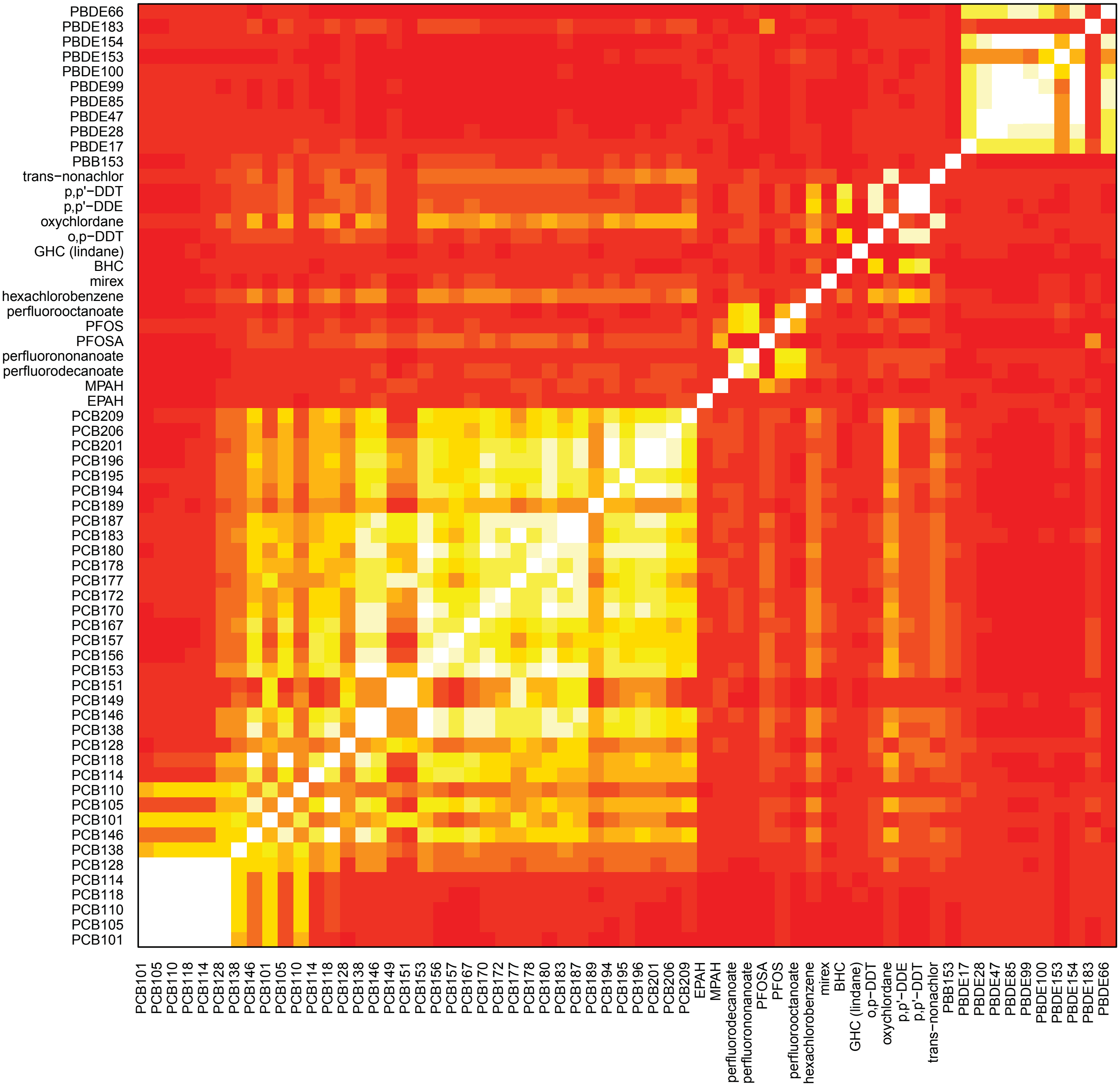

Color Scale for Correlations in the Above Heat Map

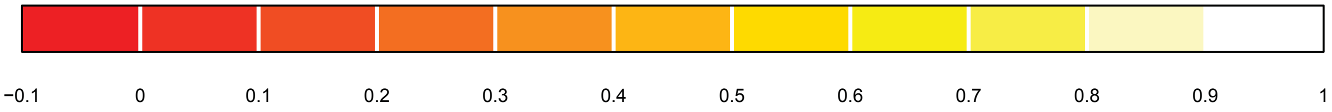

Figure S2. Cross−correlation matrix of chemicals in males against one another.

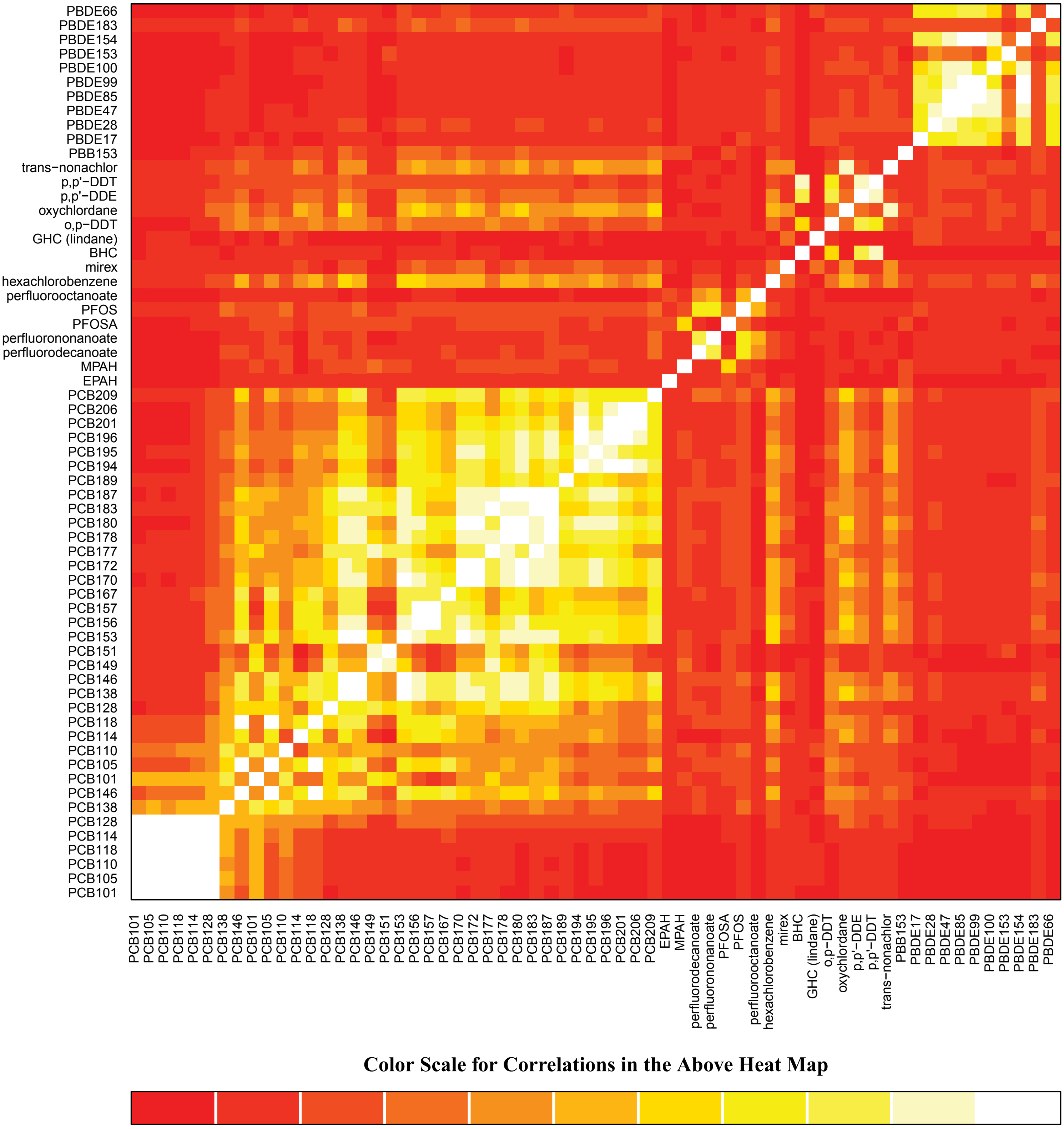

Figure S3. Cross−correlation matrix of chemicals in males against chemicals in females.

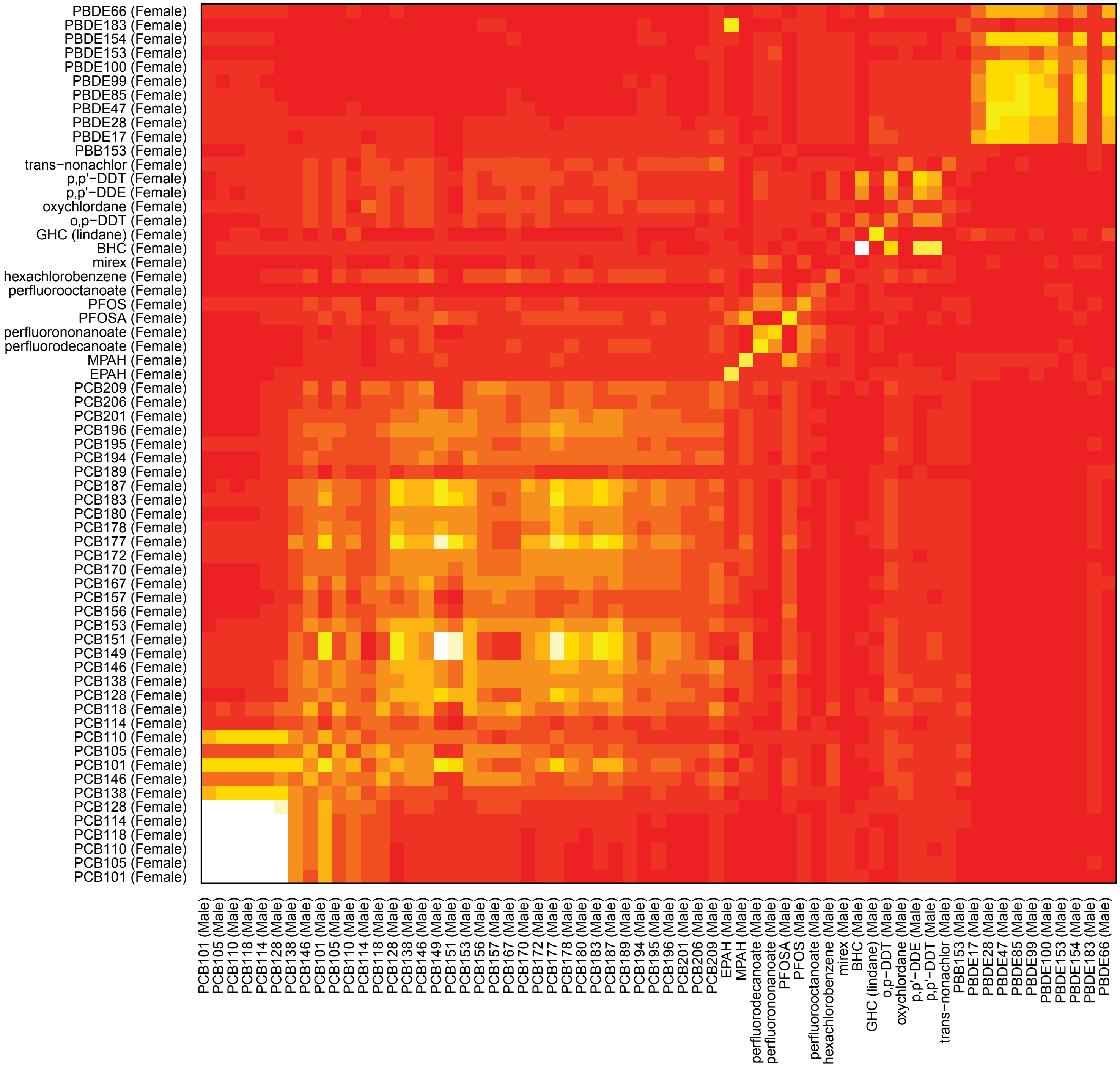

Color Scale for Correlations in the Above Heat Map

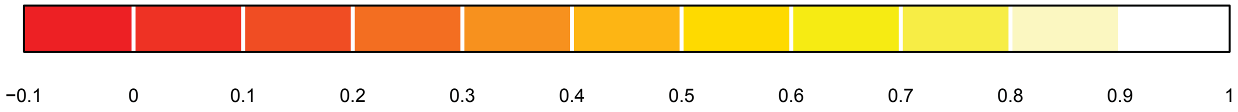

Supplement: (254 KB) PDF [file ehp.1205301.s001.pdf]
